# Effects of Supplementation of Rumen Protected Fats on Rumen Ecology and Digestibility of Nutrients in Sheep

**DOI:** 10.3390/ani9070400

**Published:** 2019-06-30

**Authors:** Atique A. Behan, Teck Chwen Loh, Sharida Fakurazi, Ubedullah Kaka, Asmatullah Kaka, Anjas Asmara Samsudin

**Affiliations:** 1 Department of Animal Science, Faculty of Agriculture, Universiti Putra Malaysia, Serdang 43400, Selangor, Malaysia; 2 Department of Livestock Management, Faculty of Animal Husbandry and Veterinary Sciences, Sindh Agriculture University, Tandojam 70060, Pakistan; 3 Department of Human Anatomy, Faculty of Medicine and Health Science, Universiti Putra Malaysia, Serdang 43400, Selangor, Malaysia; 4 Department of Clinical Studies, Faculty of Veterinary Medicine, Universiti Malaysia Kelantan, Bachok 16300, Kelantan, Malaysia; 5 Department of Animal Reproduction, Faculty of Animal Husbandry and Veterinary Sciences, Sindh Agriculture University, Tandojam 70060, Pakistan

**Keywords:** bacterial population, Dorper sheep, fatty acids, nutrient digestibility, rumen fermentation, rumen protected fat

## Abstract

**Simple Summary:**

Rising populations and urbanization are transforming into increased demand for livestock products, particularly in developing countries. The world will need more meat and more milk and in order to meet these demands, huge quantities of feed resources will be required. However, there is a substantial deficit of energy feeds affecting the growth and production of animals. The common method to increase energy value of ruminant diets is to provide them with fats. However, higher level of fats in the diet could prove toxic to rumen microbes and affect fibre digestibility, which ultimately results in reducing the feed intake and lowering animal production. These negative effects of fat supplementation can easily be overcome by feeding ruminants with specifically designed fats called rumen protected fats. In order to evaluate the efficacy of rumen protected fats (RPF), three different types of protected fats were examined in sheep. The results suggested that different types of protected fats have no unfavourable influences on the ruminal fermentation and productive parameters. Therefore, prilled fat, prilled fat with lecithin and calcium soaps did not improve animal performance as compared to the diet without protected fats in Dorper sheep.

**Abstract:**

Rumen protected fats (RPF) are known to improve animal performance without affecting rumen metabolism in sheep. However, comparative effects of prilled fat, prilled fat with lecithin and calcium soap have not been fully studied. Hence this experiment was planned using 36 male Dorper sheep in a completely randomized design in four treatment groups. The diets included: Basal diet (70:30 concentrate to rice straw) with no added RPF as a control (CON), basal diet plus prilled fat (PF), basal diet plus prilled fat with lecithin (PFL) and basal diet plus calcium soap (CaS). The trial lasted 90 days following two weeks adaptation period. The body weights, average daily gain and gain to feed ratio were not affected by treatments. The intake and digestibilities of dry matter, organic matter, crude protein and neutral detergent fibre were not affected, while those for ether extract and crude fibre differed (*p* < 0.05). RPF had no effect on concentrations of ammonia nitrogen, total volatile fatty acids and total bacterial population. The concentrations of rumen total saturated fatty acids, unsaturated fatty acids, total *n* − 3, total *n* − 6, unsaturated fatty acids:saturated fatty acids and polyunsaturated fatty acids:saturated fatty acids differed (*p* < 0.05) among the treatments with RPF supplementation. Hence supplementation of different types of protected fats did not influence animal performance in Dorper sheep.

## 1. Introduction

Rumen protected fats (RPF) are considered as insoluble fats because they are protected from microbial fermentation and biohydrogenation and remain insoluble at normal rumen pH. Thus, RPF that escape rumen fermentation are then utilized as a source of energy when absorbed through the small intestine. The fibre digestibility can be improved in high fat supplemented diets by feeding RPF [1]. Moreover, supplementation of RPF improves energy efficiency as a result of reduced production of methane from the rumen and direct use of long-chain fatty acids [2].

The studies conducted on prilled fat reported that its supplementation improved the digestibility of dry matter and organic matter [3]. It did not affect body weight of the animals in lambs. There was no change observed in dry matter intake (DMI) [4]. However, Haddad [3] reported reduction in DMI and an increase was reported by Salinas [5] with prilled supplementation in sheep.

The supplementation of calcium soap improves fibre digestibility in the fat added diets by forming insoluble soaps and protects the fatty acids from rumen degradation [1]. Ngidi et al. [6] reported that supplementation of calcium salts of fatty acids increased the digestibility of neutral detergent fibre (NDF) and digestibility of acid detergent fibre (ADF) tended to increase. Similarly, the digestibility of crude protein, ether extract and fibre fractions including ADF, NDF, cellulose and hemicellulose also increased by supplementation of calcium soap [7].

Salinas et al. [5] reported that there was a reduction observed in the digestibilities of dry matter (DM), energy, ADF and fatty acids with supplementation of lecithin in sheep. However, lecithin increased digestion of fatty acids compared to the corn oil [8]. The lecithin with protected fat improved digestion of fatty acids for 25 percent replacement of commercially available RPF [9]. It is indicated from the above discussion that there is an apparent inconsistency which might be due to the types of protected fats used resulting in a slow release of fatty acids in the rumen fluid which allowed hydrogenation of fatty acids to occur, and thus impaired microbial activity. Hence the study was planned to evaluate the influences of different types of RPF on body weight changes, nutrient intake, digestibility, rumen metabolism and rumen microbial population in Dorper sheep.

## 2. Materials and Methods

### 2.1. Location, Animals and Housing

The experiment was carried out at Department of Animal Science, Faculty of Agriculture, Universiti Putra Malaysia following the guidelines approved by the Institutional Animal Care and Use Committee (IACUC) of the Universiti Putra Malaysia (Approval Number R064/2016). Thirty-six male Dorper sheep about 18 months of age with an average initial body weight 24.66 ± 0.76 kg (mean ± SE), kept in individual wooden pens measuring 1.2 m–1 m each, built inside a shed with slatted floor 0.5 m above ground with feeding and drinking facilities were fed the experimental diet for 90 days including last 10 days for digestibility trial. A two-week adaptation period was allowed before the experiment. The animals were drenched against parasites prior to the commencement of the trial.

### 2.2. Experimental Design and Treatments

The experiment was conducted using completely randomized design (CRD) and animals were randomly allocated to four treatment groups containing nine animals in each group. The random selection was done in Microsoft Excel using tag number of animals. The rumen protected fats (RPF) (Table 1) were obtained from two different companies commercially available in the market. The four isocaloric and isonitrogenous diets, formulated to meet the nutritional requirements of sheep following the recommendations of National Research Council (NRC) [10], were: (1) basal diet (rice straw, corn starch, soybean meal, palm oil, calcium carbonate, sodium chloride, vitamin-mineral mix) with no added RPF as a control (CON); (2) basal diet plus prilled fat (PF); (3) basal diet plus prilled fat with lecithin (PFL); and (4) basal diet plus calcium soap (calcium salts of palm fatty acids) (CaS) on dry matter basis at 5% of the dry matter DM. The animals were offered feed based on their individual weight at 3% body weight on DM basis. The diets were fed a complete mix ration (rice straw and concentrate) twice daily at 0800 h and 1700 h. Water was available ad-libitum. Feed intake of each animal was recorded every day based on the quantity of feed offered and refused.

### 2.3. Chemical Analysis

Proximate analysis (dry matter, organic matter, crude protein, crude fibre) of the diets, refusal feed and the faecal samples was determined according to the protocol of Association of Official Analytical Chemists (AOAC) [11]. Neutral detergent fibre, acid detergent fibre and acid detergent lignin were determined according to the method of Van Soest et al. [12]. The fatty acid composition of the rumen protected fats is presented in Table 1. The ingredients and proximate composition of the experimental diets are presented in Table 2.

### 2.4. Body Weight Changes

The body weight of each animal was recorded on the first day of the experiment before morning feeding and every 15 days thereafter, for the 90 days experimental period to determine body weight changes.

### 2.5. Apparent Digestibility

Five animals from each treatment group were transferred to the metabolic cages provided with feeding and drinking facilities for digestibility trial. The animals were given three days as adaptation period followed by 10 days for faeces collection. The diets were fed twice every day at 0800 h and 1700 h with access to clean drinking water ad-libitum. The refusal feed was collected to calculate feed intake. Complete faecal samples were collected every day from each animal, weighed for 10 consecutive days and about 250 g samples were dried in an oven at 65 °C for 48 h to determine the DM contents. The faecal samples were ground to pass through 1 mm sieve and stored at −20 °C for proximate analysis.

The digestibility coefficient was calculated using following formula:

(1) $$\mathrm{Digestibility}\;\%=\frac{Nutrient\;consumed-Nutrient\;excreted\;in\;faeces}{Nutrient\;consumed\;\left(g/day\right)}\times 100$$

### 2.6. Rumen Liquor Collection and Fermentation Parameters

All the animals were slaughtered at Universiti Putra Malaysia abattoir by the Halal procedure, the gastrointestinal tract of each animal was removed and about 100 mL of rumen liquor was collected by sampling the rumen contents from the ventral, caudal and central areas of the rumen, pooled together and filtered using four layers of cheesecloth. The pH of rumen fluid was determined immediately after collection with Mettler-Toledo pH meter (Mettler-Toledo (M) Sdn Bhd, Shah Alam, Malaysia). Then 2 mL of 25% metaphosphoric acid was added to rumen liquor to stop the fermentation, snap-frozen in liquid nitrogen and kept at −80 °C until further analysis.

### 2.7. Determination of Volatile Fatty Acids

The frozen rumen liquor was thawed and centrifuged at 10,000 g at 4 °C for 20 min, and supernatant was collected to determine the concentrations of volatile fatty acids (VFA) The supernatant (0.5 mL) was then mixed with 0.5 mL 20 mM methyl n-valeric acid (internal standard) and was analyzed by gas chromatograph (Hewlett Packard) 6890 GC system (Agilent Technologies, Palo Alto, CA, USA) with bonded phase fused silica capillary column (15m, 0.32 mm ID, 0.25 μm film thickness) equipped with a flame ionization detector (FID). The authentic standards of acetic, propionic, butyric, isobutyric, valeric, isovaleric and 4-methyl-n-valeric acids (Sigma, St. Louis, MO, USA) were used to compare and identify the peaks.

### 2.8. Determination of Ammonia Nitrogen

Ammonia nitrogen (NH_3_-N) was determined using the colourimetric method described by Solorzano [13]. A standard curve was made to know the linear relationship between the varying concentrations of ammonium sulphate standard solution and the intensity of colour produced. This intensity was measured at a wavelength of 420 nm by a spectrophotometer (Secomam, Domont, France) within 5–10 min after placing it at 0 absorbance with the blank.

### 2.9. Fatty Acid Analysis

Fatty acids from the rumen liquor were extracted in chloroform: methanol (2:1, *v*/*v*) mixture by using the protocol of Folch et al. [14] modified by Rajion et al. [15]. The fatty acids were transmethylated into their fatty acid methyl esters (FAME) using 0.66 N KOH in methanol and 14% methanolic boron trifluoride (BF_3_) as per the procedure of Association of Official Analytical Chemists (AOAC) [11]. Heneicosanoic acid was used as the internal standard. The FAME was separated in a gas chromatograph (Agilent 7890A—Agilent Technologies, Palo Alto, CA, USA) equipped with a flame ionization detector. A reference standard (mix C4-C24 methyl esters; Sigma-Aldrich, Inc., St. Louis, MO, USA) and Conjugated Linoleic acid standard mixture (O-5507 Sigma-Aldrich, Inc., St. Louis, MO, USA) were used to determine individual FA composition.

### 2.10. Microbial Quantification

The DNA was extracted from rumen fluid using the QIAamp^®^ Fast DNA Stool Mini Kit (Qiagen, GmbH, Germany) according to manufacturer’s instructions. The quantitative real-time PCR was performed using the BioRad CFX96 Touch Real-Time PCR Detection System (BioRad, Hercules, CA, USA) by ultra-clear cap strips. The qPCR reaction was performed with the total volume of 25 µL, using the iQTMSYBR Green Supermix (BioRad). The reaction consisted of 12.5 µL SYBR Green Supermix, 1 µL of each reverse primer, 1 µL of forward primer, 2 µL of extracted DNA samples and 8.5 µL of DNAse-free water. The q-PCR conditions applied to each well were initially incubated at 94 °C for 5 min and then 40 cycles at 94 °C for 20 sec for denaturation followed by annealing temperature for 30 sec, and extended to 72 °C for 20 sec [16]. The primers used for this analysis are shown in Table 3.

### 2.11. Statistical Analysis

Statistical analysis of the data obtained was performed using generalized linear model (GLM) procedure of the SAS software 9.4 (SAS Institute Inc., Cary, NC, USA.). All data were analysed by the least-squares means method using the GLM procedures of SAS. Significantly different means were then further separated using Tukey HSD test. The microbial population data were checked for normality using the UNIVARIATE procedure of SAS software 9.4 (SAS Institute Inc., Cary, NC, USA.). All statistical tests were conducted at 95% confidence level.

## 3. Results

### 3.1. Body Weight Changes and Feed Efficiency

The body weight (BW), total weight gain, average daily gain (ADG), dry matter intake (DMI) and gain to feed ratio of the animals fed with the four dietary treatments were not affected (*p* > 0.05) among the treatment groups (Table 4).

### 3.2. Nutrient Intake and Digestibility

The intake and digestibilities of dry matter (DM), organic matter (OM), crude protein (CP), neutral detergent fibre (NDF) and acid detergent fibre (ADF) were similar. On the other hand, intake and digestibility values for ether extract (EE) and crude fibre (CF) were different (*p* < 0.05) across the treatment groups with supplementation of RPF (Table 5).

### 3.3. Rumen Fermentation Characteristics

The pH of rumen fluid was different (*p* < 0.05) across the treatments being less acidic 6.83, in the diet PFL and more acidic 6.50, in the CaS diet. The concentration of ammonia nitrogen (NH_3_-N) ranged from 14.94 mM for CaS to 16.55 mM for PFL but was not different across the treatments. Total VFA concentration was similar across the treatment groups. VFA molar proportions acetate, isobutyrate and acetate to propionate ratio were different (*p* < 0.05) while propionate and butyrate were similar among the treatments (Table 6).

### 3.4. Rumen Microbial Population

The mean concentration of total bacterial population was similar across all treatments. However, there was a difference (*p* < 0.05) in the populations of *Fibrobacter succinogenes, Ruminococcus albus* and *Ruminococcus flavefaciens* within treatments. The supplementation of RPF increased the population of *F. succinogenes* and *R. albus* significantly as compared to the diet without RPF while the population of *R. flavefaciens* and total cellulolytic bacteria was found significantly lowest in the diet CaS. The population of total protozoa was different (*p* < 0.05) being lowest in PF and highest in CaS. The population of total methanogens was not different (Table 7).

### 3.5. Fatty Acid Profile of Ruminal Digesta

For rumen digesta FA composition, no difference was observed in concentrations of C12:0, C14:0, C15:0, C15:1, C16:1*n* − 7, C16:1*n* − 9, C18:1*n* − 9 CLA *cis*-9 *trans*-11, CLA *trans*-10 *cis*-12, C20:4n − 6, C20:5*n* − 3 and C22:6*n* − 3. On the other hand, the concentrations of C16:0, C18:0, C18:1 *trans* 11, C18:2*n* − 6, C18:3*n* − 3 and C22:5*n* − 3 were influenced (*p* < 0.05) by supplementation of different RPF. The concentrations of the total saturated fatty acids (SFA), total unsaturated fatty acids (UFA), total monounsaturated fatty acids (MUFA), total polyunsaturated fatty acids (PUFA), total ∑*n* − 3, total ∑*n* − 6, UFA:SFA and PUFA:SFA differ significantly among the treatments in response to supplementation of RPF (Table 8).

## 4. Discussion

### 4.1. Body Weight Changes and Feed Efficiency

Initial and final body weights and total weight gain in the present study were similar in all the treatment groups. Similar findings were reported by Haddad and Younis [3], when they fed 21 Awassi lambs with three experimental diets; control (without RPF), 2.5% and 5% RPF respectively and found similar body weight for all lambs in three diets. Mobeen et al. [21] also found that RPF did not influence weight gain in dairy cows. Similar findings were reported for final body weight [22,23,24]. However, Bhatt et al. [7] reported that body weight in cull ewes fed RPF improved significantly (*p* < 0.05) as compared to control when they added 20 and 40 g of RPF of rice bran oil to the experimental diets. No differences in average daily gain (ADG) and gain to feed ratio found among experimental diets in the current study could be ascribed to the similar energy and protein levels of the experimental diets (Table 2). Haddad and Younis [3] reported similar ADG because of the same ME intake among all treatment groups. Manso et al. [25] reported that ADG was not affected by the treatments in lambs when they used palm oil and calcium soaps of palm oil fatty acids at two levels: 25 and 41 g fatty acids/kg respectively. However, in contrast, they found better (*p* = 0.03) feed conversion ratio (FCR) than the control diet. Contrary to the present study, Bhatt and Sahoo [26] reported improved weight gain and feed efficiency with RPF supplementation in cull ewes.

The poor feed efficiency values in the current study could be attributed to the adult animals in the present study as the age of the animals was about 18 months thus the effect of RPF could not be seen obviously when it is offered during the finishing period. Park et al. [2] reported that there was no positive difference in final body weight ADG, DMI and FCR among the treatments with amino acid-enriched rumen protected FA supplementation in steers. Gilbert et al. [27] reported similar DMI, ADG and FCR values in steers by adding protected canola lipid to the diets, and Ngidi et al. [6] reported that DMI, ADG and FCR in steers were not affected by supplementing with 2% calcium salts of FA. However, it was reported by Haaland et al. [28] and Reddy et al. [29] that RPF had no negative influence on DMI, ADG and FCR.

### 4.2. Nutrient Intake

Supplementation of RPF did not influence daily feed, DM, OM, NDF, ADF and ME intakes. The outcome of the current study is in agreement with Mudgal et al. [30] who found no influence of RPF supplementation on DMI. Similarly, Salinas et al. [5] reported that different levels of Ca soaps of tallow fed to lambs had no effect on DMI. Park et al. [2] also reported similar findings for DMI in steers. There was no decrease in DMI when several vegetable fats were added in the ration of lambs in different studies [4,31,32,33,34]. On the other hand, Bhatt et al. [35] reported increased feed and nutrient intake with supplementation of calcium salts of FA in Malpura lambs. The main factor restricting DMI is dietary NDF content [36] and when forages are the main source of NDF, the intake of DM and NDF are negatively correlated with each other. In the present study, the forage fibre used was rice straw. Although the diets had different concentrations of rice straw, NDF contents were similar among the diets, which could also be the reason for similar intakes in the present study.

In contrast to the current study, Haddad and Younis [3] found a reduction in DMI after including 25 and 50 g/kg of saturated fat in the diets of Awassi lamb. Lough et al. [37] also noticed a reduction in DMI when 100 g/kg palm oil was added to the diet. The study conducted by Vandoni et al. [38] contradicts the present study to the extent that in their study DMI was higher in animals given unprotected fat compared with animals given calcium soap but the present study reported no significant difference in DMI. Their findings are similar to the extent that calcium soap reduced DMI compared to other diets so also in the present study CaS diet has not only reduced feed intake but also EE intake. In a review paper Allen [39] it is reported that increasing concentrations of Ca salts of palm FA had lower DMI in 22 out of 24 studies reported. There could be a possible reason for DMI reduction in the CaS in the present study as the feeding of calcium soaps to animals causes apparent palatability problems followed by an ultimate reduction in DMI [6,40]. However, the RPF containing calcium soap in the current study was mixed very well with the concentrate by using feed mixer but the reduction in dry matter intake could possibly be the effect of calcium soap as several previous studies also reported palatability problem with calcium soap supplementation. Variable results on palatability were reported as no change in palatability [4], decreased DMI [3], and increased DMI [5] with calcium soap supplementation.

The intake of ME was not affected by the supplementation of RPF. These findings are supported by Schauff et al. [41] and Haddad and Younis [3] who found that ME intake was not altered by adding RPF to the experimental diets. In contrast, Bhatt and Sahoo [26] reported higher ME intake with RPF supplementation. One possible reason for lack of significant differences in ME intake among the treatment groups in the present study could be low forage contents in the diets. It was reported that the supplementation of fat to a low-forage diet did not influence ADG and DMI, while supplementation of fat to a high-forage diet improved ADG in finishing steers [42].

### 4.3. Apparent Nutrient Digestibility

Supplementation of RPF did not influence DM, OM, CP, NDF and ADF digestibilities in Dorper sheep. This is consistent with studies conducted by Naik et al. [43], who found that feeding RPF to buffaloes had no influence on the digestibilities of DM, OM, CP, total carbohydrates and NDF. Similar effects of RPF supplementation on digestibilities were reported by several studies [44,45,46]. In contrast, Bhatt and Sahoo [26] reported higher OM and EE digestibilities with RPF supplementation. Schauff and Clark [47] stated that there was an increase in CP digestibility with supplementation of the calcium salt of long-chain fatty acid to the dairy animals.

The reduction in DM digestibility in diets with RPF compared to the diet without RPF is consistent with most findings [6,8,48] while it is in contrast with the findings of Haddad and Younis [3] who found an increase in the DM digestibility as fat addition increased.

The present study also contradicts with Hightshoe et al. [49] who reported higher DM digestibility in beef cattle fed various commercially available RPF. This difference could be due to the level of protected fat used as they used 90 g/kg of total diet while in the present study the level was 50 g/kg of diet. They suggested an upper limit to the inclusion of calcium salts of fatty acids when they observed that overall DM and ADF digestion were decreased only when the salts were included at 90 g/kg of total diet. However, there was no reduction in DM or fibre digestibility observed with supplementation of calcium salts of fatty acids in lactating dairy cows [47] and in steers [6].

The EE and CF digestibilities differ significantly among treatment groups in the present study. Animals fed PFL and PF had the highest EE and CF digestibilities. These results could be attributed to the emulsifying properties and lipid digestion enhancement from lecithin in PFL. Hence, lipids are rendered insoluble in the rumen and absorbed easily after emulsification. The increase in EE digestibility can be ascribed to high-quality fat inclusion in the diets [50]. Another study conducted by Voigt et al. [51] reported increased EE digestibility values with supplementation of RPF. Similar findings were reported by studies conducted by [7,44,46,52]. It was also reported that improved digestibility of EE and CF with supplementation of calcium salts was because of increased digestion of FA in Ca soap as a result of the lower ruminal biohydrogenation (BH) and consequently higher concentrations of UFA in intestinal chime [26]. In general, unprotected fat that passes through rumen is prone to lipolysis and biohydrogenation. The digestibility of fatty acid increases as unsaturation increases. Hence, the higher digestibility of EE and CP in RPF diets might be due to the lower ruminal biohydrogenetion and availability of large proportion of long chain unsaturated fatty acids in the small intestine for absorption [53].

Conversely, CaS yielded the poorest result for EE digestibility. This could be because of the FA composition, as CaS contained the highest concentration of unsaturated C18 FA and the lowest amount of saturated C18 FA. Alexander et al. [53] reported decreased EE digestibility in the diet containing calcium soap as compared to the diet without calcium soap. Another study also reported apparently lower EE digestibility when sheep were fed calcium soaps at 12% of the diet compared to those animals fed at 6% of the diet [54]. Hence, the fat intake was relatively similar in all three RPF diets but different concentration of unsaturated C18 FA could have affected the results for apparent digestibility of EE in CaS.

The increased digestibility of CP in the PFL and CaS is in agreement with findings of Bhatt et al. [7] who found increased CP digestibility with RPF supplementation. The microbial protein synthesis might have been influenced by the decrease in readily fermentable carbohydrates in the rumen which causes loss of NH_3_-N [55] and lower quantities of starch available at large intestine [56]. There is microbial growth in the large intestine which shows up as CP in the faeces that could have been responsible for lower apparent CP digestibility.

The apparent digestibility of ADF in the PFL and CaS was significantly affected by supplementation of RPF when compared to the diet without RPF (CON). Naik et al. [43] also reported an increase in ADF digestibility with RPF supplementation. The ADF digestibility changes with the amount of calcium salt of long chain fatty acid supplementation in the diet and is not affected at a low level of supplementation [57]. Type of FA of calcium salt affects the digestibility of ADF. The increase in unsaturation of dominant FA of calcium salt improves the digestibility of ADF quadratically [58].

### 4.4. Rumen Fermentation Characteristics

The similar rumen fermentation parameters including NH_3_-N, total VFA and molar proportions of VFA are consistent with a study reporting that rumen pH, NH_3_-N and VFA levels were not affected adversely by the supplementation of calcium soaps up to five percent of the DM in lactating cows [59].

The rumen pH values in the present study are different (*p* < 0.05) across the treatment groups, and are in agreement with the findings reported by Cheng et al. [60] who reported lowered pH at 4 h post feeding and Wang et al. [61], who reported that pH was decreased quadratically with increased rumen protected folic acid supplementation in beef steers while in present study in PF and CaS the pH values decreased and in PFL pH values increased in comparison to the diet without RPF (CON). In contrast, the pH values did not differ significantly across the treatment groups in several previous studies [6,7,38,47,49,62,63,64]. The pH was highest in PFL diet and lowest in CaS diet. These highest and lowest pH values in PFL and CaS respectively accorded well with the total VFA concentration as pH is inversely proportional to total VFA concentration [65]. The reason for the variation in the pH values could be the different feeding pattern of animals [6].

The concentrations of total VFA were similar in the present study throughout the treatments which is supported by Hightshoe [49], who reported that neither source nor level of RPF influenced both total VFA and acetate-to-propionate ratio in steers fed calcium soaps and hydrogenated animal fats. Similarly, no differences were observed in rumen pH and VFA concentration by feeding three different types of RPF [34]. Hence these studies support the similar VFA concentration in the present study. In contrast, total VFA concentration was slightly lower in PFL compared to all other treatments in the present study, which could possibly be the effect of lecithin in the diet as the inclusion of lecithin might have reduced energy available to the bacteria decreasing microbial protein synthesis and VFA production.

In PFL, molar proportions of acetate reduced and molar proportions of propionate increased, ultimately reducing acetate to propionate ratio. Similar findings were reported by Schauff et al. [41] who observed a reduction in total VFA and acetate with the increase in propionate which decreased acetate to propionate ratio with supplementation of tallow and whole soybean. The higher acetate, lower propionate molar proportions and higher acetate to propionate ratio in the CaS diet could possibly be the effect of calcium in the present study. Similar findings were observed by Schauff and Clark [47], who reported an increment in acetate and reduction in propionate ultimately resulting in a linear increase in acetate to propionate ratio with increasing level of calcium salts of long chain fatty acids fed to the cattle.

The concentration of NH_3_-N was not altered (*p* > 0.05) with supplementation of RPF among the treatment groups. Similar findings were observed by the studies conducted by [7,41,47,53,57]. In contrast, a study reported that supplementation of RPF in beef cattle increased NH_3_-N levels [53]. The pH and VFA production follow the opposite trend, and on the other hand, NH_3_-N and protozoa number follow a similar trend [66]. The similar and non-significant results of the rumen fermentation parameters including, NH_3_-N, total VFA, molar proportions propionate and butyrate indicate that all RPF were inert in the rumen, hence had no major influence on rumen fermentation when supplemented at current levels.

### 4.5. Rumen Microbial Population

Diet is one of the key factors that influence rumen microbial populations [31,67]. The wide range of microorganisms including cellulolytic, proteolytic and amylolytic bacteria, anaerobic fungi and protozoa occupy rumen and play an important role in the digestion of fibre and other nutrients present in feed components [68,69]. The fermentation by rumen microbes generates VFA and the subsequent microbial mass is a potential source of protein that can be digested and absorbed by the host for growth [70]. However, the proportions of bacteria, protozoa and fungi in the rumen are influenced by diet composition, and often set the limit for biomass production and feed use efficiency [71].

The population of total bacteria did not differ significantly in the present study, which could be attributed to similar concentrate diets in all the treatment groups and is supported by the findings of rumen fermentation profile.

The significant difference (*p* < 0.05) in rumen protozoa population was observed among the treatments being the maximum increase seen in the CaS diet. Similar to the findings of the present study, Ca soap supplementation increased protozoa population in cull ewes [7]. The reduction seen in protozoa in PF diet along with a significant increase in *Ruminococcus albus* is explained by the fact that the reduction of protozoa in the rumen often results in greater proliferation of bacteria and greater passage of bacterial nitrogen N out of the rumen [72]. The population of total methanogens was similar throughout all the diets which is supported by the findings of Beauchemin et al. [73] who reported that RPF have no influence on methanogenesis and digestibility of nutrients.

The *Ruminococcus albus, Ruminococcus flavefaciens* and *Fibrobacter succinogenes* are the most predominant cellulolytic species in the rumen, which obtain nutrients by breaking down cellulose that comes through the digestive system of the host organism and as such, changes in their relative amounts could potentially affect ruminal fiber metabolism and concentrations of volatile fatty acids [74]. There was a significant increase observed in the populations of *F. succinogenes* and *R. albus* with RPF supplementation as compared to the diet without RPF. The reduction in these two cellulolytic bacteria in CON could be the effect of palm oil present in the diet as oil supplementation is known to exert a toxic effect on cellulolytic bacteria and to ultimately reduce fibre digestibility [75,76]. The decrease in *R. albus* in the present study is also supported by an in vitro study in which there was a reduction observed in the population of *R. albus* with oil supplementation [16].

The PF diet had a significantly higher population of *R. albus* as compared to other treatments. This could be due to the reduction in the population of rumen protozoa. In the present study, the CaS decreased significantly the population of *R*. *flavefaciens* and total cellulolytic bacteria could be because CaS contained the highest concentration of UFA (Table 2) since the high proportion of UFA is toxic to rumen microbial populations and particularly to cellulolytic bacteria [77].

### 4.6. Rumen Fatty Acid Profile

The fatty acids released in the rumen are not absorbed by the rumen microbes, and are passed to the abomasum then the small intestine, which is the primary site for absorption of the fatty acids [78]. The main FA in highest concentration seen in rumen digesta of sheep fed RPF was stearic acid (C18:0) in all treatment groups, which is obvious as C18:0 is the end product of rumen BH [79]. RPF supplementation decreased the production of C18:0 in rumen fluid significantly (*p* < 0.05) in PF, PFL and CaS compared to CON diet. This reduction of C18:0 with RPF supplementation shows that the RPF were inert in nature, therefore, could not go under complete BH by rumen microbes as compared to CON which gave the highest concentration of C18:0. Another reason for higher C18:0 concentrations in the rumen digesta of the sheep fed diet without RPF could be that CON contained higher amount of oleic acid (C18:1*n* − 9), linoleic acid (C18:2*n* − 6) and linolenic acid (C18:3*n* − 3) comparative to RPF diets, thus higher intake of these FA caused higher C18:0 concentration in rumen of sheep fed CON diet due to BH process [80].

The lowest concentration of C18:0 was observed in PF with the higher concentrations of BH intermediates CLA *cis*-9 *trans*-11 and C18:1 *trans* 11, this could be the result of incomplete BH of C18:2*n* − 6, C18:3*n* − 3 and C18:1*n* − 9. Similar findings were reported by several studies that incomplete BH of C18:2*n* − 6, C18:3*n* − 3 and C18:1*n* − 9 FA yielded CLA *cis*-9 *trans*-11, CLA *cis*-12 *trans*-10 and C18:1 *trans* 11 intermediate isomers [81,82,83]. The current study is supported by the results of Kim et al. [84] who found higher concentrations of C18:0 in rumen digesta of lambs fed diets with lower *n* – 6 ÷ *n* − 3 ratio, similarly in the present study there was the highest concentration of C18:0 in CON which had lower *n* – 6 ÷ *n* − 3 ratio. These findings could be because of the differences in the nature of dietary FA used, which in the present study was RPF. There were no differences found in concentrations of CLA among the diets with RPF supplementation. This could be because the source of fat was protected fat. Gulati et al. [85] performed a study in vitro on the effect of incubating protected and unprotected CLA under anaerobic conditions at 38 °C for 24 h. A high percentage of unprotected CLA was hydrogenated to trans-vaccenic acid compared with protected CLA while protected CLA was not hydrogenated.

The second highest concentration of FA found in rumen digesta of sheep was palmitic acid (C16:0) because the dietary treatments contained higher amounts of C16:0 and all RPF also had more than 70 percent of C16:0 concentration. The concentration of C16:0 in rumen digesta of sheep fed with rumen protected fat was higher in PF, PFL and CaS in comparison to CON; this was expected firstly because of the chemical composition of the diets which were high in C16:0, and secondly, because of the inertness of RPF.

The concentrations of CLA *cis*-9 *trans*-11 and CLA *trans*-10 *cis*-12 were not different significantly (*p* > 0.05) throughout the treatment groups. The lowest concentration of CLA *cis*-9 *trans*-11 was observed in CON. Similarly, Schmidely et al. [86] reported the low duodenal flow of CLA *cis*-9 *trans*-11 and CLA *trans*-10 *cis*-12 in goats fed 45 g/day of calcium salts of palm oil and 45 g/day of lipid-encapsulated conjugated linoleic acids. However, the factors that affect the concentration of CLA isomers are the fat content and polyunsaturated FA concentration of the diet, the percentage of concentrate and interaction between these two factors [81,87].

## 5. Conclusions

The findings of the current study indicate that PF, PFL and CaS supplementation did not have a negative impact on body weight changes, nutrient intake, digestibility and rumen fermentation parameters. However, the RPF diets did not show significant differences when compared to the CON diet. Therefore, supplementation of different types of protected fats did not influence animal performance in Dorper sheep.

## Figures and Tables

**Table 1:** Fatty acid composition of rumen protected fats.

Fatty Acids (% of Total FA)	PF ^1^	PFL ^1^	CaS ^1^
C15:0	1.39	1.15	1.43
C16:0	72.98	76.72	48.31
C16:1*n* − 9	0.16	0.05	0.81
C18:0	5.16	4.92	4.33
C18:1*n* − 9	16.34	12.85	41.15
C18:2*n* − 6	3.40	3.94	1.64
C18:3*n* − 3	0.57	0.37	2.33
ΣSFA ^2^	79.53	82.79	54.07
ΣMUFA ^2^	16.5	12.90	41.95
ΣPUFA ^2^	3.97	4.31	3.97
*n* – 6 ÷ *n* − 3 ^2^	5.96	10.65	0.70

^1^ PF—Prilled fat; ^1^ PFL—Prilled fat with lecithin; ^1^ CaS—Calcium soap of palm fatty acids; ^2^ Calculated ΣSFA—Total saturated fatty acid (C15:0 + C16:0 + C18:0), ^2^ ΣMUFA—Total monounsaturated fatty acid (C16:1*n* − 9 + C18:1*n* − 9), ^2^ ΣPUFA = Total polyunsaturated fatty acid (C18:2*n* − 6 + C18:3*n* − 3) ^2^ *n* − 6 ÷ *n* − 3 = (C18:2*n* − 6 ÷ C18:3*n* − 3).

**Table 2:** Ingredients, chemical and fatty acid composition of experimental diets.

	Diets			
Ingredient (%)	CON ^1^	PF ^1^	PFL ^1^	CaS ^1^
Rice straw (urea treated)	29	33	34	35
Corn starch	39	31	30	29
Soybean meal	26	27	27	27
Palm oil	4	2	2	2
Calcium carbonate	1	1	1	1
Sodium chloride	0.5	0.5	0.5	0.5
Vitamin-mineral mix ^2^	0.5	0.5	0.5	0.5
Prilled fat (RPFA) ^3^	-	5	-	-
Prilled fat with lecithin (RPFB)	-	-	5	-
Calcium soap (RPFC)	-	-	-	5
Total	100	100	100	100
**Chemical composition (% DM)**				
Dry Matter (DM)	91.54	92.08	92.31	91.95
Organic Matter (OM)	93.29	91.23	91.31	91.96
Ether Extract (EE)	4.98	8.27	8.04	8.38
Crude Protein (CP)	18.88	18.57	19.92	19.23
Crude Fibre (CF)	12.67	28.42	30.09	13.77
Neutral Detergent Fibre (NDF)	56.06	58.78	60.22	51.32
Acid Detergent Fibre (ADF)	17.89	21.80	24.33	23.94
Acid Detergent Lignin (ADL)	6.65	13.33	10.35	4.92
ME (MJ/kg DM) ^4^	11.68	11.65	11.66	11.68
**Fatty acids (% of total FA)**				
C15:0	0.78	1.12	0.95	0.60
C16:0	32.93	59.52	61.49	21.21
C16:1*n* − 9	0.20	0.13	0.11	0.30
C18:0	3.78	4.87	4.58	2.62
C18:1*n* − 9	42.38	24.20	21.83	52.70
C18:2*n* − 6	18.93	9.69	10.44	21.51
C18:3*n* − 3	1.00	0.47	0.60	1.06
ΣSFA ^5^	37.49	65.51	67.03	24.43
ΣMUFA ^5^	42.58	24.33	21.94	53.00
ΣPUFA ^5^	19.93	10.16	11.04	22.56
*n* – 6 ÷ *n* − 3 ^5^	18.93	20.62	17.40	20.29

^1^ CON—Basal diet without RPF; ^1^ PF—Basal diet + prilled fat; ^1^ PFL—Basal diet + prilled fat with lecithin; ^1^ CaS—Basal diet + calcium soap; ^2^ Contained (g/kg) CuSO_4_·5H_2_O, 70; ZnSO_4_·7H_2_O, 240; FeSO_4_·7H_2_O, 170; MnSO_4_·5H_2_O, 290; (mg/kg) CoCl_2_·6H_2_O 510; KI, 220; NaSeO, 130; vitamin B1, 450; pantothenic acid, 750; vitamin K3, 150; folic acid, 15; vitamin B12, 0.9; vitamin B5, 1050; (IU), vitamin D3, 324,000; vitamin A, 620,000; ^3^ RPFA—Rumen protected fat-A (prilled fat); RPFB – Rumen protected fat-B (prilled fat with lecithin); RPFC – Rumen protected fat-C (calcium soap); ^4^ ME - Metabolizable energy; ^5^ Calculated = ΣSFA—Total saturated fatty acid (C15:0 + C16:0 + C18:0); ^5^ ΣMUFA—Total monounsaturated fatty acid (C16:1*n* − 9 + C18:1*n* − 9), ^5^ ΣPUFA = Total polyunsaturated fatty acid (C18:2*n* − 6 + C18:3*n* − 3) ^5^ *n* − 6 ÷ *n* − 3 = (C18:2*n* − 6 ÷ C18:3*n* − 3).

**Table 3:** Primers used for quantitative real-time polymerase chain reaction (q-PCR).

Microorganism		Sequence 5′–3′	Product Size (bp)	Annealing Temperature °C	Reference
Total bacteria	F	CGGCAACGAGCGCAACCC	145	55	1
	R	CCATTGTAGCACGTGTGTAGCC			
Total protozoa	F	CTTGCCCTCYAATCGTWCT	223	55	2
	R	GCTTTCGWTGGTAGTGTATT			
Total methanogens	F	CCGGAGATGGAACCTGAGAC	160	55	3
	R	CGGTCTTGCCCAGCTCTTATTC			
*Fibrobacter succinogenes*	F	GTTCGGAATTACTGGGCGTAAA	122	55	4
	R	CGCCTGCCCCTGAACTATC			
*Ruminococcus albus*	F	CCCTAAAAGCAGTCTTAGTTCG	175	55	1
	R	CCTCCTTGCGGTTAGAACA			
*Ruminococcus flavefaciens*	F	TCTGGAAACGGATGGTA	259	58	1
	R	CCTTTAAGACAGGAGTTTACAA			

F—forward; R—reverse; 1—Koike and Kobayashi [17]; 2—Sylvester et al. [18]; 3—Zhou and Hernandez-Sanabria [19]; 4—Lane [20].

**Table 4:** Effect of rumen protected fat on body weight changes, average daily gain and gain:feed ratio in Dorper sheep.

Parameter	Treatments					
	CON ^1^	PF ^1^	PFL ^1^	CaS ^1^	SEM ^2^	*p* Value
Initial BW ^3^ (kg)	24.73	24.63	24.83	24.58	0.825	0.999
Final BW ^3^ (kg)	35.06	35.18	35.42	34.04	0.759	0.930
Total WG ^4^ (kg)	10.32	10.68	10.59	9.47	0.332	0.576
ADG (g) ^5^	117.30	121.34	120.33	107.58	3.778	0.576
DMI (g/day) ^6^	896.83	895.17	903.83	879.33	4.261	0.987
Gain:Feed	0.135	0.141	0.136	0.128	0.007	0.874

^1^ CON—Basal diet without RPF; PF—Basal diet + prilled fat; PFL—Basal diet + prilled fat with lecithin; CaS—Basal diet + calcium soap; ^2^ SEM—standard error of means; ^3^ BW—body weight; ^4^ WG—weight gain; ^5^ ADG—average daily gain; ^6^ DMI—dry matter intake.

**Table 5:** Effect of different rumen protected fats on daily average nutrient intake and digestibility in Dorper sheep.

Nutrient Intake (g/day)			Treatments			
	CON ^1^	PF ^1^	PFL ^1^	CaS ^1^	SEM ^2^	*p* Value
Daily feed	982.22	972.16	979.13	954.96	5.091	0.985
DM ^3^	896.83	895.17	903.83	879.33	4.261	0.987
OM ^3^	865.13	836.22	842.80	827.58	7.192	0.953
EE ^3^	49.06 ^b^	80.45 ^a^	78.54 ^a^	35.36 ^c^	3.681	<0.0001
CP ^3^	161.78 ^b^	180.75 ^ab^	195.78 ^a^	183.31 ^ab^	5.872	0.054
CF ^3^	123.97 ^b^	277.16 ^a^	294.33 ^a^	131.68 ^b^	14.908	<0.0001
NDF ^3^	551.94	571.72	589.27	489.17	15.892	0.126
ADF ^3^	252.07	215.16	238.32	230.03	7.298	0.350
ADL ^3^	67.27 ^c^	129.38 ^a^	100.76 ^b^	48.22 ^c^	6.811	<0.0001
**Apparent digestibility (%)**						
DM	76.86	71.98	76.27	75.96	1.272	0.577
OM	89.38	88.37	89.94	87.64	0.447	0.348
EE	50.43 ^ab^	63.19 ^a^	66.78 ^a^	33.94 ^b^	4.565	0.013
CP	48.78	47.47	58.34	55.34	2.533	0.411
CF	43.04 ^b^	71.80 ^a^	74.81 ^a^	54.72 ^b^	4.446	0.006
NDF	61.63	58.13	63.17	58.87	1.851	0.803
ADF	36.31	30.93	52.88	58.36	4.981	0.147

^a, b, c,^ means having different superscript in each row are significantly different (*p* < 0.05); ^1^ CON—Basal diet without RPF; PF—Basal diet + prilled fat; PFL—Basal diet + prilled fat with lecithin; CaS—Basal diet + calcium soap; ^2^ SEM—standard error of means; ^3^ DM—dry matter; OM—organic matter; EE—Ether extract; CP—crude protein; CF—crude fibre; NDF—neutral detergent fibre; ADF—acid detergent fibre; ADL—acid detergent lignin.

**Table 6:** Effect of different rumen protected fats on rumen fermentation characteristics in Dorper sheep.

Parameter			Treatments			
	CON ^1^	PF ^1^	PFL ^1^	CaS ^1^	SEM ^2^	*p* Value
Rumen pH	6.61 ^b^	6.60 ^b^	6.83 ^a^	6.50 ^b^	0.038	0.012
NH_3_-N (mg/dL)	16.14	15.12	16.55	14.94	0.330	0.246
Total VFA (m*M*)	46.41	47.07	43.44	47.84	1.357	0.703
Molar proportion (%)						
Acetate (A)	42.02 ^ab^	44.51 ^a^	40.32 ^b^	44.28 ^a^	0.595	0.028
Propionate (P)	30.99	29.02	34.14	28.32	0.869	0.070
Butyrate (B)	17.25	18.36	15.32	18.82	0.502	0.056
Isobutyrate	9.74 ^ab^	8.11 ^b^	10.22 ^a^	8.59 ^ab^	0.314	0.050
A + B/P	1.94 ^ab^	2.19 ^a^	1.66 ^b^	2.35 ^a^	0.091	0.030
P ÷ TVFA ^3^	0.31	0.29	0.34	0.28	0.009	0.074
A:P	1.37 ^ab^	1.56 ^a^	1.20 ^b^	1.64 ^a^	0.061	0.039

^a, b, c,^ means having different superscript in each row are significantly different (*p* < 0.05); ^1^ CON—Basal diet without RPF; PF—Basal diet + prilled fat; PFL—Basal diet + prilled fat with lecithin; CaS—Basal diet + calcium soap; ^2^ SEM—standard error of means; ^3^ TVFA—total volatile fatty acid.

**Table 7:** Effect of different rumen protected fats on microbial population (copies/mL) in the rumen of Dorper sheep.

Microorganism			Treatments			
(Log ^10^ Cells/mL)	CON ^1^	PF ^1^	PFL ^1^	CaS ^1^	SEM ^2^	*p* Value
Total bacteria	8.40	8.64	8.62	8.33	0.057	0.119
Total protozoa	5.86 ^a^	3.80 ^b^	5.21 ^a^	6.02 ^a^	0.256	0.001
Total methanogens	5.92	5.93	5.62	5.94	0.054	0.095
*Fibrobacter succinogenes*	5.82 ^c^	6.61 ^a^	6.63 ^a^	6.33 ^b^	0.132	0.048
*Ruminococcus albus*	6.92 ^c^	8.33 ^a^	7.71 ^b^	7.11 ^c^	0.136	<0.0001
*Ruminococcus flavefaciens*	5.62 ^a^	5.35 ^a^	5.99 ^a^	3.65 ^b^	0.202	0.001
Total cellulolytic bacteria	18.37^b^	20.29^a^	20.34^a^	17.09^b^	0.304	<0.0001

^a, b, c,^ means having different superscript in each row are significantly different (*p* < 0.05); ^1^ CON—Basal diet without RPF; PF—Basal diet + prilled fat; PFL—Basal diet + prilled fat with lecithin; CaS—Basal diet + calcium soap; ^2^ SEM—standard error of means.

**Table 8:** Effect of different rumen protected fats on the fatty acid composition of rumen digesta in Dorper sheep.

Parameter			Treatments			
	CON ^1^	PF ^1^	PFL ^1^	CaS ^1^	SEM ^2^	*p* Value
C12:0	1.62	1.73	1.37	1.78	0.100	0.498
C14:0	3.12	3.75	2.99	3.99	0.171	0.107
C15:0	1.13	0.94	0.68	0.93	0.097	0.452
C15:1	2.93	2.68	2.44	2.69	0.113	0.515
C16:0	24.91 ^b^	31.86 ^a^	30.90 ^a^	31.57 ^a^	0.613	<0.0001
C16:1*n* − 7	0.53	0.44	0.38	0.57	0.059	0.697
C16:1*n* − 9	1.78	1.82	2.22	1.63	0.107	0.255
C18:0	50.41 ^a^	41.94 ^c^	46.01 ^b^	42.22 ^c^	0.757	<0.0001
C18:1*n* − 9	3.87	3.53	3.52	3.47	0.311	0.231
C18:1t11	6.23 ^b^	9.78 ^a^	6.31 ^b^	5.90 ^b^	0.336	<0.0001
CLA C9 T11	0.38	0.51	0.48	0.44	0.043	0.740
CLA T10 C12	0.63	0.48	0.52	0.51	0.059	0.843
C18:2*n* − 6	0.97 ^b^	1.35 ^b^	2.05 ^a^	1.78 ^a^	0.103	<0.0001
C18:3*n* − 3	0.33 ^b,c^	0.28 ^c^	1.09 ^a^	0.78 ^ab^	0.099	0.004
C20:4*n* − 6	0.29	0.54	0.49	0.59	0.051	0.192
C20:5*n* − 3	0.20 ^b^	0.40 ^a,b^	0.55 ^a^	0.37 ^a,b^	0.049	0.078
C22:5*n* − 3	0.15 ^c^	0.53 ^a,b^	0.56 ^a^	0.28 ^b,c^	0.055	0.010
C22:6*n* − 3	0.52	0.45	0.45	0.51	0.053	0.941
∑SFA ^3^	81.19 ^b^	80.22 ^c^	81.95 ^a^	80.84 ^b,c^	0.170	<0.0007
∑UFA ^3^	18.81 ^b^	19.79 ^a^	18.05 ^c^	19.16 ^a,b^	0.169	<0.0006
∑MUFA ^3^	15.35 ^a^	15.25 ^a^	11.86 ^c^	14.80 ^b^	0.281	<0.0001
∑PUFA ^3^	3.46 ^c^	4.53 ^b^	6.19 ^a^	4.36 ^b^	0.221	<0.0001
∑*n* − 3 ^3^	1.20 ^c^	1.66 ^b,c^	2.65 ^a^	1.57 ^b^	0.127	<0.0001
∑*n* − 6 ^3^	1.26 ^c^	1.88 ^b^	2.54 ^a^	1.82 ^a,b^	0.125	<0.0001
*n* − 6 ÷ *n* − 3 ^3^	1.11	1.21	1.01	1.21	0.083	0.625
UFA:SFA ^3^	0.23 ^b^	0.25 ^a^	0.22 ^c^	0.24 ^b^	0.002	<0.0008
PUFA:SFA ^3^	0.04 ^c^	0.06 ^b^	0.08 ^a^	0.05 ^b^	0.003	<0.0001

^a, b, c,^ means having different superscript in each row are significantly different (*p* < 0.05); ^1^ CON—Basal diet without RPF; PF—Basal diet + prilled fat; PFL—Basal diet + prilled fat with lecithin; CaS—Basal diet + calcium soap; ^2^ SEM—standard error of means; ^3^ ΣSFA = (C12:0 + C14:0 + C15:0 + C16:0 + C18:0), ΣUFA = (ΣMUFA + ΣPUFA), ΣMUFA= (C15:1 + C16:1*n* − 7 + C16:1*n* − 9 + C18:1*n* − 9 + C18:1 *trans*-11), ΣPUFA= (ΣCLA + Σ*n* − 3 + Σ*n* − 6), Σ*n* − 3= (C18:3*n* − 3 + C20:5*n* − 3 + C22:5*n* − 3 + C22:6*n* − 3), Σ*n* − 6 = (C18:2*n* − 6 + C20:4*n* − 6), *n* − 6 ÷ *n* − 3 = (C18:2*n* − 6 + C20:4*n* − 6) ÷ (C18:3*n* − 3 + C20:5*n* − 3 + C22:5*n* − 3 + C22:6*n* − 3), UFA:SFA = ((ΣMUFA + ΣPUFA) ÷ ΣSFA), PUFA:SFA = (ΣPUFA÷ΣSFA).
